# Detrimental health relationship between blood lead and cadmium and the red blood cell folate level

**DOI:** 10.1038/s41598-022-10562-9

**Published:** 2022-04-22

**Authors:** Bo-kai Wang, Wei-Liang Chen

**Affiliations:** 1 Department of Anesthesiology, Tri-Service General Hospital, School of Medicine, National Defense Medical Center, Taipei, Taiwan, ROC; 2 Department of Internal Medicine, Tri-Service General Hospital, School of Medicine, National Defense Medical Center, Taipei, Taiwan, ROC; 3 Division of Family Medicine, Department of Family and Community Medicine, Tri-Service General Hospital, School of Medicine, National Defense Medical Center, Taipei, Taiwan, ROC; 4 Division of Geriatric Medicine, Department of Family and Community Medicine, Tri-Service General Hospital, School of Medicine, National Defense Medical Center, Number 325, Section 2, Chang-gong Rd, Nei-Hu District, Taipei, 114 Taiwan, ROC; 5 Department of Biochemistry, National Defense Medical Center, Taipei, Taiwan, ROC

**Keywords:** Metals, Biomarkers, Medical research

## Abstract

Increasing studies have demonstrated the association between heavy metal pollution and micronutrients, especially folate. However, the relationship between cadmium and folate remains rarely discussed. In this study, we aim to explore the potential correlation between cadmium and folate in human population and highlight the possible mechanism of cadmium impacting human health. We utilized the National Health and Nutrition Examination Survey (NHANES) 2017–2018 data with 5690 participants in this study. Multivariable linear regression models were adopted to investigate the serum lead and cadmium levels and RBC folate concentration. A significant reverse relationship was found between serum lead and cadmium and RBC folate. A negative relationship between serum lead and cadmium levels and the levels of RBC folate in the U.S. adult population was found in this study. Nevertheless, due to the general limitations of the NHANES data, as a cross-sectional study, a further prospective investigation is needed to discover the causality of lead and cadmium in folate status and to determine whether the folate supplement has a beneficial influence against heavy metal toxicities.

## Introduction

Along with flourishing industrialization, the heavy metal pollution contributed by them has become a health issue in urban populations. More than 10 kinds of metallic chemical elements or metalloids are included in heavy metal pollution^1^. Among them, cadmium (Cd) plays an important role in affecting the human health. Cd can result in various major adverse effects, involving kidney disease, bone disease, lung disease, and several cancers^2^. Furthermore, in recent studies, cadmium is found to cause excess oxidative stress, dysregulation of the miRNA expression, modulation of cellular signaling pathways, deregulation of cell proliferation, and apoptosis resistance^3^. The effects of heavy metal pollution on micronutrients have also been increasingly reported^4–6^.

Folates, known as vitamin B9, are water-soluble vitamins that are not able to be synthesized by the human cells and are naturally present in food. They form a crucial part of one-carbon metabolism, involved in the synthesis of DNA, RNA, and proteins. In addition, evidence has also suggested that folates have antioxidant, anticancer, cardiovascular, and neuroprotective effects^7^. The health advantage of folates have been well recognized, and since 1998, even the U.S. Government has enforced mandatory fortification of all cereal grains containing folic acids to prevent neural tube defects during pregnancy. Red blood cell (RBC) folate is one of the biomarkers to represent folate levels in the individual. It is specific in its expression of tissue folate levels, which would not be affected by temporary changes in dietary intake^8^. As a result, it is a useful surrogate of long-term folate status instead of serum folate concentration.

A rising number of research studies disclose the robust relationship between toxic heavy metals and homocysteine metabolism^9^. Homocysteine is an intermediate amino acid in the conversion of methionine and cysteine, and the accumulation of it will cause adverse heath effects. As a necessary cofactor, folate plays an essential part in homocysteine metabolism. However, the role of heavy metal pollution, especially cadmium, in folate status in human remained undefined. Hence, this article aimed to investigate the connection between cadmium levels and levels of folate using data from the National Health and Nutrition Examination Survey (NHANES) from 2017 to 2018.

## Materials and methods

### Ethics statement

All data in this study were obtained from the National Health and Nutrition Examination Survey (NHANES) database, which was authorized by the National Center for Health Statistics (NCHS) of the Centers for Disease Control and Prevention (CDC). The NHANES database was approved by the NCHS Institutional Review Board (Protocol #2018-01 and Continuation of Protocol #2011-17) in accordance with the revised Helsinki Declaration. The informed consents forms were completed before the data collection procedures and extensive health examinations.

### Data source and participants

The data were extracted from the NHANES from 2017 to 2018, which contained a comprehensive interview and a series of examination. The survey is a cross-sectional study executed by the National Center for Health Statistics (NCHS) of the Centers for Disease Control and Prevention (CDC), aiming to explore the health and nutritional status of noninstitutionalized U.S. civilians. All details and information of NHANES are available on the website. We analyzed a wide range of materials, including demographic data, questionnaire details, laboratory results, RBC folate levels, and heavy metal levels. Participants with missing associated information or related covariates were excluded. A total of 5690 participants were included in this study.

### Measurement of blood lead and cadmium

The blood lead and cadmium levels were detected via an inductively coupled plasma mass spectrometry with dynamic reaction cell (ICP-DRS-MS). The limits of detection for blood lead and blood cadmium were 0.07 µg/dL and 0.1 µg/L, respectively. In healthy, unexposed adults, concentrations of blood cadmium are in the range of 0.1–4 µg/L. In this study, the inter-assay coefficient of variation for lead and cadmium were 2% and 4.93%, sequentially.

### Measurement of red blood cell folate

In NHANES, RBC folate was calculated from the whole blood folate concentration, which was detected in microbiological assays with inoculated medium containing *Lactobacillus rhamnosus*^10^. The whole blood folate concentration was evaluated by estimating the turbidity of the *L. rhamnosus* medium using the PowerWave X340 Microplate reader (Bio-Tek Instrument) at a wavelength of 590 nm wave. Next, whole blood hemolysate folate results, detected by the microplate reader, were multiplied by 11, the dilution factor of the whole blood. The serum folate values, which was accessed by isotope-dilution high performance liquid chromatography/tandem mass spectrometry (LC–MS/MS), were subtracted, and the resulting value was divided by the hematocrit (Hct) to convert RBC folate into nmol/L RBC. In this LC–MS method, five folate forms would be detected, including folic acid, tetrahydrofolate, 5-methyl-tetrahydrofolate, 5-formyl-tetrahydrofolate, and 5,10-methenyl-tetrahydrofolate^11^. A multi-rule quality control program was adopted in this study. The reference range of RBC folate is 505–2,490 nmol/L. Besides, the inter-assay coefficient of variation is 5.1%.

### Covariates

Some of related variables, including age, gender, race/ethnicity, smoking status, and liver condition, were obtained by self-report. Smoked participants were identified whenever they self-reported having smoked at least 100+ cigarettes during their lifetime. The liver condition was classified through the question “Do you still have a liver condition?” Participants’ platelet counts were measured by the Beckman Coulter DxH-800 analyzer in the NHANES Mobile Examination Center. The urinary albumin levels were assessed using a fluorescein immunoassay by the Sequoia-Turner Digital Fluorometer, Model 450 by University of Minnesota. Other biochemistry profiles, including alanine transaminase (ALT), serum creatinine, and serum total bilirubin, were attained with Roche Cobas 6000 analyzer performed by the University of Minnesota. All the protocols followed the standardized procedures of the CDC reference method and were available on the NHANES websites.

### Statistical analysis

All the statistical investigations were conducted using the Statistical Package for the Social Sciences software, version 18.0 (SPSS Inc., Chicago, IL, USA). Categorical variables were presented by their means and standard deviation (SD), whereas continuous variables were presented by their frequency counts and percentages. We analyzed categorical features with the Chi-square test and continuous features with the Wilcoxon Rank sum test, respectively. Using multivariable logistic regression models, an extended-model investigation was designed for covariate adjustment as follows: Model 1 was not adjusted for any variables; Model 2 was adjusted for age, sex and race/ethnicity; Model 3 = Model 2 + platelet count, urinary albumin, ALT, total bilirubin, and creatinine; Model 4 = Model 3 + history of smoking. A two-sided *p*-value of less than 0.05 was interpreted as statistically significant.

## Results

### Heavy metal and RBC folate

The association between circulating concentrations of heavy metals and RBC folate concentration of the 5690 enrolled participants is shown in Table 1. In a fully adjusted model, the β coefficient between lead, cadmium, mercury, manganese, selenium, and RBC folate are − 18.278 (95% CI − 25.835 to − 10.721; *p* < 0.001), − 119.261 (95% CI − 210.556, − 27.965; *p* = 0.011), − 2.745 (95% CI − 5.513 to 0.022; *p* = 0.052), 4.401 (95% CI 2.416–6.387; *p* < 0.001), and 0.478 (95% CI 0.18–0.776; *p* = 0.002), respectively. These findings demonstrate that lead and cadmium have a significant reverse correlation with RBC folate concentration, while manganese and selenium have a significantly positive correlation with RBC folate concentration. Further analysis of the relationship between lead and cadmium and different folate forms is revealed in Tables S1 and S2, respectively. The results illustrate that both blood lead and blood cadmium have a significant reverse associated with serum total folate, 5-methyl-tetrahydrofolate, and tetrahydrofolate.

**Table 1:** Association between circulating concentrations of heavy metal and RBC folate concentration.

Trace elements	Model 1 β (95% CI)	*p*	Model 2 β (95% CI)	*p*	Model 3 β (95% CI)	*p*	Model 4 β (95% CI)	*p*
Lead (ug/dL)	2.092(− 5.182, 9.366 )	0.573	− 19.109 (− 26.659, − 11.558)	< 0.001	− 19.033 (− 26.563, − 11.503)	< 0.001	− 18.278 (− 25.835, − 10.721)	< 0.001
Cadmium (ug/L)	− 119.409 (− 203.556, − 35.263)	0.006	− 112.071 (− 197.129, − 27.013)	0.01	− 113.361 (− 199.393, − 27.33)	0.01	− 119.261 (− 210.556, − 27.965)	0.011
Mercury (ug/L)	− 1.577 (− 4.424, 1.27)	0.278	− 2.561 (− 5.324, 0.202)	0.069	− 2.456 (− 5.219, 0.307)	0.081	− 2.745 (− 5.513, 0.022)	0.052
Manganese (ug/L)	2.007 (0.01, 4.003)	0.049	4.666 (2.703, 6.63)	< 0.001	4.559 (2.577, 6.541)	< 0.001	4.401 (2.416, 6.387)	< 0.001
Selenium (ug/L)	0.51 (0.2, 0.821)	0.001	0.486 (0.188, 0.783)	0.001	0.479 (0.181, 0.778)	0.002	0.478 (0.18, 0.776)	0.002

*RBC* red blood cell, *CI*, confidence interval.

β coefficients were interpreted as change of RBC folate concentration for each increase in different trace elements concentration.

Adjusted covariates: Model 1 = Unadjusted model; Model 2 = Adjusted for age, sex and race/ethnicity; Model 3 = Model 2 + platelet count, urinary albumin, ALT, total bilirubin, and creatinine; Model 4 = Model 3 + history of smoked (defined as ever smoked at least 100 cigarettes in a lifetime).

### Demographic data of Study population

Tables 2 and 3 illustrate the demographic characteristics of the participants categorized by lead and cadmium tertiles, respectively. The tertile-based lead levels among the study population were < 0.57 µg/dL, 0.57–1.13 µg/dL, > 1.13 µg/dL, while the tertile-based cadmium levels among the study population were < 0.19 µg/L, 0.19–0.39 µg/L, and > 0.39 µg/L, correspondingly. The participants with higher lead or cadmium levels tended to be older and smokers, and they had higher levels of urinary albumin and creatinine and lower levels of platelet count. The participants with higher lead levels were more likely to be males and had higher levels of total bilirubin, whereas the participants with higher cadmium levels were more likely to be females and had lower levels of total bilirubin.

**Table 2:** Characteristics of study participants by tertiles of serum lead concentration (µg/L).

	T1 (< 0.57 µg/dL) (n = 1917)	T2 (0.57–1.13 µg/dL) (n = 1876)	T3 (> 1.13 µg/dL) (n = 1897)	Total (n = 5690)	*p*
Serum Lead (µg/L)	0.39 (0.11)	0.84 (0.16)	2.21 (1.91)	1.14 (1.35)	< 0.001
Age (years)	30.06 (16.06)	47.57 (19.12)	57.49 (16.7)	44.98 (20.87)	< 0.001
Platelet count (1000 cells/uL)	261.59 (61.73)	244.25 (63.73)	233.97 (62.03)	246.67 (63.52)	< 0.001
Albumin, urine (ug/mL)	35.81 (164.94)	45.85 (286.35)	64.7 (463.62)	48.7 (328.03)	0.023
ALT (IU/L)	20.92 (19.69)	22.14 (15.85)	21.85 (15.39)	21.63 (17.1)	0.074
Creatinine (mg/dL)	0.77 (0.19)	0.88 (0.37)	0.97 (0.62)	0.87 (0.44)	< 0.001
Total bilirubin (mg/dL)	0.44 (0.29)	0.46 (0.28)	0.48 (0.27)	0.46 (0.28)	0.001
Male	38.4	50.9	59.2	49.4	< 0.001
Non-Hispanic White	32.7	33.7	34.8	33.7	< 0.001
Liver condition	1.25	3.09	3	2.44	0.198
Smoked^a^	16.74	38.33	50.61	35.15	< 0.001

^a^“Smoked” was defined as ever smoked at least 100 cigarettes in a lifetime.

**Table 3:** Characteristics of study participants by tertiles of serum cadmium concentration (µg/L).

	T1 (< 0.19 µg/L) (n = 1950)	T2 (0.19 µg/L–0.39 µg/L) (n = 1858)	T3 (> 0.39 µg/L) (n = 1882)	Total (n = 5690)	*p*
Serum Cadmium (µg/L)	0.12 (0.04)	0.28 (0.06)	0.91 (0.73)	0.43 (0.54)	< 0.001
Age (years)	34.04 (19.77)	48.25 (20.3)	53.08 (17.49)	44.98 (20.87)	< 0.001
Platelet count (1000 cells/uL)	252.5 (58.0)	243.93 (62.76)	243.33 (69.1)	246.67 (63.52)	< 0.001
Albumin, urine (ug/mL)	32.28 (137.51)	52.14 (406.49)	62.44 (377.94)	48.7 (328.03)	0.016
ALT (IU/L)	22.01 (16.52)	21.64 (16.01)	21.23 (18.67)	21.63 (17.1)	0.37
Creatinine (mg/dL)	0.83 (0.22)	0.88 (0.42)	0.92 (0.59)	0.87 (0.44)	< 0.001
Total bilirubin (mg/dL)	0.47 (0.3)	0.47 (0.29)	0.44 (0.25)	0.46 (0.28)	< 0.001
Male	59.8	45.6	42.4	49.4	< 0.001
Non-Hispanic White	34.7	32.8	33.6	33.7	< 0.001
Liver condition	1.28	2.1	3.99	2.44	0.082
Smoked^a^	15.02	29.98	61.11	35.15	< 0.001

^a^“Smoked” was defined as ever smoked at least 100 cigarettes in a lifetime.

### Relationships between levels of RBC folate, lead, and cadmium levels

**Figure 1:**
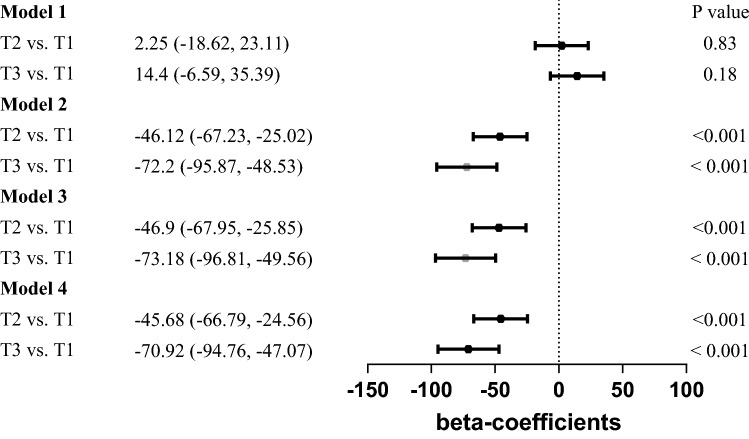
β-coefficient (95% CI) for RBC folate concentration by tertile of serum lead concentration.

**Figure 2:**
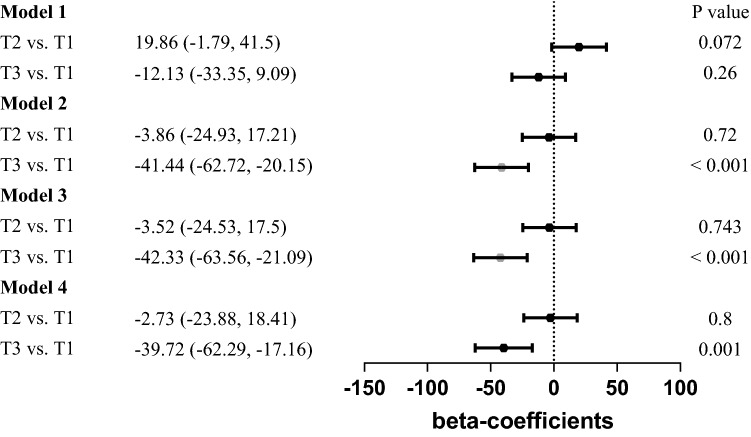
β-coefficient (95% CI) for RBC folate concentration by tertile of serum cadmium concentration.

We examined the association between RBC folate and lead and cadmium levels. The result is shown in Figs. 1 and 2. In the tertiles-based multiple linear regression analysis, RBC folate levels in participants with T3 cadmium were significantly lower than those with T1 cadmium in all adjusted models. It revealed the same finding in the analysis of tertiles of lead. The β coefficients between the T3 lead group and the T1 lead group in all models were 14.4(95% CI − 6.59 to 35.39; *p* = 0.18 in model 1), − 72.2(95% CI − 95.87 to − 48.53; *p* < 0.001 in model 2), − 73.18(95% CI − 96.81 to − 49.56; *p* < 0.001 in model 3), and − 70.92(95% CI − 94.76 to − 47.07; *p* < 0.001 in model 4), correspondingly, while the β coefficients between the T3 cadmium group and the T1 cadmium group in all models were − 33.35(95% CI − 12.13 to 9.09; *p* = 0.26 in model 1), − 41.44(95% CI − 62.72 to − 20.15; *p* < 0.001 in model 2), − 42.33(95% CI − 63.56 to − 21.09; *p* < 0.001 in model 3), and − 39.72(95% CI − 62.29 to − 17.16; *p* = 0.001 in model 4), respectively. Collectively, the above findings indicated that serum lead and cadmium levels had a significantly negative correlation with RBC folate levels.

## Discussion

In this study of the US general population, the most remarkable finding was that serum lead and cadmium levels both had an adverse relationship with RBC folate levels. The association became stronger when the highest tertile group was compared with the lowest tertile group. To the best of our knowledge, our research is the first report investigating the relationship between cadmium levels and body folate status.

After reviewing the published articles, a few studies explored the association between lead or cadmium and folate. In animal model, folic acid supplementation seemed to have potentially protective effects against lead exposure in rat pups^12^. In a multicenter prospective Korean study, containing 2171 participants, serum folate levels in pregnant women were reversely associated with blood cadmium concentrations during late pregnancy^13^. Additionally, two small-scale studies reported that higher blood lead or cadmium levels might be associated with higher levels of serum homocysteine^14,15^. In a cohort with 259 participants, Pollack et al. found that in healthy, premenopausal women, blood lead and cadmium were correlated with increased levels of homocysteine, but this correlation was not strong after adjustment for corresponding confounding factors in cadmium group. Meanwhile, it was also not significant among those consuming ≧ 75th percentile of vitamin B_6_, vitamin B_12_, and folate. A Chinese-population-based study, containing 159 individuals, indicated that increasing blood lead and cadmium levels could raise the probability of a surge in homocysteine levels. Although the relationship between lead and cadmium and homocysteine was not robustly proven, there was still a positive trend. Many research studies have illustrated that homocysteine levels were inversely related to serum folate^16–18^. In one systemic study, Ledda et al. found a similar result, showing that exposure to heavy metal, including lead and cadmium, is correlated with homocysteine levels or folate serum concentrations^9^. Besides, the role of red cell folate in presenting an individual’s folate status has been well established^8^. All the above mentioned data are consistent with our findings. Unfortunately, the populations of the studies mentioned above were all too narrow to apply their results to the general population. Our investigation provided more general and large-scale evidence on this issue.

Cadmium has been proven to cause numerus adverse effects in humans via several pathways, including increased oxidative stress, impairment of the antioxidant response, enzyme inactivation, and multiple organ damage^3^. However, the mechanism between cadmium and folate status was unclear, and we tried to propose several possible hypotheses for it. First of all, cadmium was found to be absorbed via metal transporters in the intestinal, accumulating in the intestinal as cadmium exposure increased^19^, and rising animal studies found that cadmium would cause gut microbiome damage^20–22^. On the other hand, folate is primarily absorbed from the diet through the intestinal tract, and oxidative stress may interfere with the gut microbiome, resulting in folate deficiency^23^. They implied that cadmium accumulation in the gut might cause gut microbiota damage via ROS generation and increased oxidative stress, consequently, resulting in folate malabsorption. Second, Cd^2+^ was a predominant and competitive inhibitor of the enzyme^24^, and it could bind to numerus protein with the sulfhydryl group(-SH), leading to protein inactivation. Cadmium might interfere with the enzymes of the folate cycle, and it needs more studies to investigate the details. Third, it was well recognized that several heavy metals exert their toxicity through similar pathways. As for epigenetic effect, cadmium and arsenic (As) were both correlated with DNA methylation, and specific histone modification marks^25–28^. Abuawad et al. summarized the important relationship between one-carbon metabolism and arsenic methylation. Folate, as a crucial factor in one-carbon metabolism, has been proven to improve arsenic methylation capacity and to further promote arsenic elimination^29,30^. Although the mechanism of the elimination of cadmium was unclear, it might have had a similar pathway between one-carbon metabolism and cadmium. Finally, cadmium has been proven to be carcinogenic, and it could affect several transcription factors, such as activator protein 1 (AP-1), nuclear factor-kappa B (NF-κB), and p53. Hence, uncontrolled cell growth and division would happen due to a failure in the control of the expression of protective genes^31^. It might increase the requirement for folate due to abnormally increasing cell proliferation, and this hypothesis requires more research to confirm it.

Several potential limitations should be considered for our study. First, the NHANHES database is a cross-sectional study. Consequently, a causal relationship between cadmium levels and folate status could not be determined only according to this one-off health check-up program, instead of to long-standing observation study. Second, the intake of folate was not analyzed in our study. Although the dietary questionnaire used in the NHANES survey was documented, the 24-h dietary recall may not provide an indication of long-term diet nor an accurate representation of actual intake. Moreover, in spite of adjusting for multiple potentially confounding factors, there may have been other residual effects from unadjusted confounding factors of the correlation between lead and cadmium levels and folate status.

## Conclusion

This study highlighted that a negative relationship between serum lead and cadmium levels and levels of RBC folate in the U.S. adult population.

## Supplementary Information

Supplementary Tables.
