# Supplementary material for: Detrimental health relationship between blood lead and cadmium and the red blood cell folate level
Source: Sci Rep. 2022 Apr 22;12:6628. doi: 10.1038/s41598-022-10562-9 (PMC9033805; doi:10.1038/s41598-022-10562-9)
Supplement: Supplementary file 1 — Supplementary Tables. [file 41598_2022_10562_MOESM1_ESM.docx]

**Table S1. Association between blood lead and folate forms**

| **Folate forms** | **Model 1** | | **Model 2** | | **Model 3** | | **Model 4** | |
| --- | --- | --- | --- | --- | --- | --- | --- | --- |
|  | **β**  **(95% CI)** | ***P***  **Value** | **β**  **(95% CI)** | ***P***  **Value** | **β**  **(95% CI)** | ***P***  **Value** | **β**  **(95% CI)** | ***P***  **Value** |
| **Serum total folate** | 0.11 (-0.736, 0.956) | <0.001 | -1.969 (-2.851, -1.087) | <0.001 | -1.956 (-2.837, -1.074) | <0.001 | -1.788 (-2.671, -0.905) | <0.001 |
| **5-Methyl-tetrahydrofolate** | 0.095 (-0.618, 0.808) | 0.794 | -1.734 (-2.471, -0.996) | <0.001 | -1.725 (-2.46, -0.99) | <0.001 | -1.542 (-2.277, -0.807) | <0.001 |
| **Folic acid** | -0.006 (-0.308, -0.296) | 0.969 | -2.03 (-0.533, 0.126) | 0.227 | -0.198 (-0.528, 0.131) | 0.238 | -0.216 (-0.547, 0.116) | 0.202 |
| **5-Formyl-tetrahydrofolate** | -0.002 (-0.023, 0.018) | 0.83 | -0.006 (-0.029, 0.016) | 0.581 | -0.006 (-0.029, 0.016) | 0.58 | -0.006 (-0.028, 0.017) | 0.629 |
| **Tetrahydrofolate** | 0.022 (0.003, 0.040) | 0.02 | -0.023 (-0.042, -0.004) | 0.019 | -0.024 (-0.043, -0.005) | 0.015 | -0.022 (-0.041, -0.003) | 0.023 |
| **5, 10-Methenyl-tetrahydrofolate** | 0.001 (-0.003, 0.004) | 0.689 | -0.003 (-0.007, 0.001) | 0.106 | -0.03 (-0.007, 0.001) | 0.115 | -0.003 (-0.007, <0.001) | 0.087 |
| **Mefox oxidation product** | 0.109 (0.052, 0.166) | <0.001 | -0.006 (-0.068, 0.055) | 0.842 | -0.021 (-0.071, 0.03) | 0.427 | -0.02 (-0.071, 0.031) | 0.438 |

Abbreviations: CI, confidence interval.
β coefficients were interpreted as change of blood lead concentration for each increase in different folate forms concentration.

Adjusted covariates:

Model 1 = Unadjusted

Model 2 = Model 1 + age, sex, race/ethnicity

Model 3 = Model 2 + platelet count, urinary albumin, ALT, creatinine, total bilirubin

Model 4 = Model 3 + smoking

**Table S2. Association between blood cadmium and folate forms**

| **Folate forms** | **Model 1** | | **Model 2** | | **Model 3** | | **Model 4** | |
| --- | --- | --- | --- | --- | --- | --- | --- | --- |
|  | **β**  **(95% CI)** | ***P***  **Value** | **β**  **(95% CI)** | ***P***  **Value** | **β**  **(95% CI)** | ***P***  **Value** | **β**  **(95% CI)** | ***P***  **Value** |
| **Serum total folate** | -3.648 (-5.36, -1.937) | <0.001 | -4.879 (-6.531, -3.227) | <0.001 | -4.769 (-6.422 -3.116) | <0.001 | -4.126 (-5.910, -2.343) | <0.001 |
| **5-Methyl-tetrahydrofolate** | -3.595 (-5.037, -2.153) | <0.001 | -4.729 (-6.107, -3.351) | <0.001 | -4.602 (-5.978, -3.225) | <0.001 | -3.787 (-5.27, -2.303) | <0.001 |
| **Folic acid** | 0.007 (-0.606, 0.62) | 0.982 | -0.066 (-0.687, 0.555) | 0.835 | -0.083 (-0.704, 0.539) | 0.794 | -0.255 (-0.926, 0.415) | 0.455 |
| **5-Formyl-tetrahydrofolate** | -0.01 (-0.053, 0.032) | 0.627 | -0.013 (-0.055, 0.03) | 0.563 | -0.014 (-0.056, 0.029) | 0.537 | -0.008 (-0.055, 0.038) | 0.721 |
| **Tetrahydrofolate** | -0.045 (-0.082, -0.009) | 0.015 | -0.065 (-0.101, -0.029) | <0.001 | -0.064 (-0.1, -0.029) | <0.001 | -0.065 (-0.103, -0.026) | 0.001 |
| **5, 10-Methenyl-tetrahydrofolate** | -0.003 (-0.011, 0.004) | 0.353 | -0.006 (-0.013, 0.001) | 0.11 | -0.006 (-0.014, 0.001) | 0.096 | -0.01 (-0.018, -0.002) | 0.014 |
| **Mefox oxidation product** | 0.087 (-0.03, 0.203) | 0.146 | 0.048 (-0.068, 0.165) | 0.416 | 0.041 (-0.055, 0.136) | 0.405 | 0.05 (-0.053, 0.153) | 0.342 |

Abbreviations: CI, confidence interval.
β coefficients were interpreted as change of blood cadmium concentration for each increase in different folate forms concentration.

Adjusted covariates:

Model 1 = Unadjusted

Model 2 = Model 1 + age, sex, race/ethnicity

Model 3 = Model 2 + platelet count, urinary albumin, ALT, creatinine, total bilirubin

Model 4 = Model 3 + smoking
